# Hexarelin Protects Rodent Pancreatic Β-Cells Function from Cytotoxic Effects of Streptozotocin Involving Mitochondrial Signalling Pathways *In Vivo* and *In Vitro*

**DOI:** 10.1371/journal.pone.0149730

**Published:** 2016-02-26

**Authors:** Yan Zhao, Xinli Zhang, Jiezhong Chen, Chao Lin, Renfu Shao, Chunxia Yan, Chen Chen

**Affiliations:** 1 Institute of Basic Medicine Science, Xi'an Medical University, Xi'an, China; 2 Department of Forensic Science, School of Medicine, Xi’an Jiaotong University, Xi’an, China; 3 School of Biomedical Sciences, The University of Queensland, St Lucia, QLD, Australia; 4 Gene Cology Research Centre, Faculty of Science, Health, Education and Engineering, University of the Sunshine Coast, Maroochydore, QLD, Australia; Columbia University, UNITED STATES

## Abstract

Mitochondrial functions are crucial for pancreatic β-cell survival and glucose-induced insulin secretion. Hexarelin (Hex) is a synthetic small peptide ghrelin analogue, which has been shown to protect cardiomyocytes from the ischemia-reperfusion process. In this study, we used *in vitro* and *in vivo* models of streptozotocin (STZ)-induced β-cell damage to study the protective effect of Hex and the associated mechanisms. We found that STZ produced a cytotoxic effect in a dose- and time-dependent manner in MIN6 cells (a mouse β-cell line). Hex (1.0 μM) decreased the STZ-induced damage in β-cells. Rhodamine 123 assay and superoxide DHE production assay revealed that Hex ameliorated STZ-induced mitochondrial damage and excessive superoxide activity in β-cells. In addition, Hex significantly reduced STZ-induced expression of cleaved Caspases-3, Caspases-9 and the ratio of pro-apoptotic protein Bax to anti-apoptotic protein Bcl-2 in MIN6 cells. We further examined the *in vivo* effect of Hex in a rat model of type 1 diabetes induced by STZ injection. Hex ameliorated STZ-induced decrease in plasma insulin and protected the structure of islets from STZ-induced disruption. Hex also ameliorated STZ-induced expression of cleaved Caspase-9 and the Bax in β-cells. In conclusion, our data indicate that Hex is able to protects β-cell mass from STZ-caused cytotoxic effects involving mitochondrial pathways *in vitro* and *in vivo*. Hex may serve as a potential protective agent for the management of diabetes.

## Introduction

Diabetes mellitus, a global public health problem that affects in excess of 350 million people worldwide [1], is now emerging as a pandemic and by the year 2025, three quarters of the world’s 300 million adults with diabetes will be in non-industrialized countries, and almost a third in India and China alone [2]. There are two classical main types of diabetes. (1) Type 1 diabetes (T1DM) is an autoimmune disease that results in the loss of pancreatic β-cell function and hence the loss of insulin production, and accounts for about 5–10% of the population diagnosed with diabetes [3]. (2) Type 2 diabetes (T2DM) is the most common form of diabetes, accounting for >90% of the cases which result from the development of insulin resistance. T2DM, although once associated with adults and hence the older terminology of “adult onset diabetes”, is now frequently also seen in adolescents and children, and the incidence has dramatically increased worldwide [4]. The common feature of both T1DM and T2DM is the loss of glycaemic control and β-cell dysfunction [5]. Moreover, the progressive worsening of T2DM in humans is thought to result from a gradual loss of functional β-cell mass [6]. Thus, there is strong interest in dissecting the molecular pathways that lead to the decline in mass and function of β-cells in diabetes, especially as the disease remains a serious public health challenge with limited numbers of effective therapies to reverse the pathology [7].

Streptozotocin (STZ) is a widely used chemical for the induction of experimental diabetes in rodents [8, 9]. It has been used alone or in combination with other chemicals or with dietary manipulations for induction of either T1DM or T2DM [10, 11]. All these STZ-involved diabetic animal models have been very useful in elucidating the mechanisms of diabetic pathogenesis and in screening chemicals, and pharmacological agents that are potentially capable of lowering blood glucose levels [12, 13]. STZ contains a glucose molecule (in deoxy form) that is linked to a highly reactive methylnitrosourea moiety that is thought to exert STZ’s cytotoxic effects, while the glucose moiety directs the chemical to the pancreatic β-cells [14]. STZ recognizes the GLUT2 (Slc2A2) receptor which is much more abundant on β-cell plasma membranes than on other cell types (liver, kidney, small intestine) [9]. Therefore, pancreatic β-cell is accepted as a specific target of STZ. Once transported into the cell through Slc2A2 transporters, STZ kills cells by forming a toxic DNA adduct and cuntributes to mitochondrial glucotoxicity in β-cells [15, 16]. Mitochondria are essential for β-cell survival and glucose-induced insulin secretion [17–19]. Production of ATP by the mitochondria stimulates insulin exocytosis in β-cells [20]. It has also been reported that environmental and industrial pollutants can cause the death of β-cells through mitochondrial impairment [21, 22]. Thus, protection against mitochondrial dysfunction in β-cells might serve as a target in the development of novel therapeutic strategies for diabetes [23–25].

Ghrelin is a hormone mainly produced in the stomach, but also in other organs such as pancreas, which has both central and peripheral effects [26]. It acts to increase growth hormone (GH) secretion and food intake in mammals *via* the GH secretagogue-receptor 1a (GHS-R1a) [27]. In the peripheral system, ghrelin acts on various cell types including β-cells and adipocytes. In high-fat-fed mice, deletion of ghrelin or its receptor genes results in improved glucose tolerance, and enhanced insulin secretion and sensitivity [28–31]. In humans, infused ghrelin induces acute insulin resistance [32]. However, Des-acyl ghrelin does not bind to GHS-R1a and has a stimulatory effect on glucose and lipid metabolism through unknown receptors [33]. In addition, both acyl-ghrelin and des-acyl ghrelin have a positive effect on β-cell survival [34, 35]. It is still inconclusive on the influence of ghrelin on pancreatic β-cells.

Hex is a synthetic analogue of ghrelin that shows cardio-protective effects both *in vivo* and *in vitro* (via GHS-R1a) [36, 37]. It is chemically more stable and functionally more potent when compared with ghrelin [38], which makes Hex a promising substitute for ghrelin in the clinical applications. In this report, we studied the *in vitro* and *in vivo* effects of Hex on impaired β-cells following the treatment with STZ. Our findings indicate that Hex protects β-cells from STZ-induced cell dysfunction by maintaining the mitochondrial functions.

## Materials and Methods

### Chemicals and reagents

Hex was purchased from China Peptides (Shanghai, China). Reagents and consumables used for cell culture were obtained from Invitrogen (Melbourne, Victoria, Australia). STZ and other chemicals were obtained from Sigma-Aldrich (Sigma Chemical, St. Louis, MO, USA).

### Cell culture

The mouse pancreatic α-cell line (α-TC6 cells, kindly provided by Y. Moriyama, Okayama University, Okayama, Japan) and β-cell line (MIN6 cells, kindly provided by Dr. M Garry, Monash University, Clayton, Australia with the approval of Dr. J. Miyazaki, Osaka University, Osaka, Japan) were maintained in a 5% CO_2_ incubator at 37°C. α-TC6 cells were cultured in high glucose Dulbecco's modified Eagle's medium (DMEM) containing 4.5 g/L D-glucose, supplemented with 10% fetal bovine serum and 1% Penicillin-Streptomycin. MIN6 cells were cultured in the same medium with the addition of 50 μM β-mercaptoethanol.

### Haematoxylin-eosin (H&E) staining

MIN6 cells grown on 2×2 cm^2^ coverslips to 80~90% confluence were serum-starved for 6 hours. Cells were then treated with various concentrations of STZ (0, 0.5, 1.0, and 2.0 mM) for 0, 2, or 6 hours. After the treatments, cells were fixed and subjected to H&E staining as described previously [39].

### Cell treatment protocols

For investigating the protective effect of Hex on STZ-treated MIN6 cells, cells were subjected to different treatments: 1) Control group: cells kept in serum-free medium (SFM) for 4 hours followed by a 2-hour incubation in refreshed SFM with appropriate vehicles; 2) STZ treatment group: cells treated with 1 mM STZ in SFM for 4 hours followed by a 2-hour incubation in refreshed SFM; 3) Hex treatment group: cells kept in SFM for 4 hours followed by a 2-hour incubation with 1 μM Hex in SFM; and 4) “STZ + Hex” treatment group: cells treated with 1 mM STZ in SFM for 4 hours followed by a 2-hour incubation with 1 μM Hex in SFM.

### Cell viability assay

MIN6 and α-TC6 cells were seeded in 96-well plates and cultured in the presence or absence of Hex (1 μM) and STZ (1 mM) for 0.5, 1, 2, 4, and 6 hours. After the treatment, cells were replaced in fresh SFM and the number of viable cells was quantified according to the manufacturer’s protocols using CellTiter 96 Aqueous One Solution Cell Proliferation Assay (Promega, Madison, WI, USA), which contains the tetrazolium compound [3-(4,5-dimethylthiazol2-yl)-5-(3-carboxy- methoxyphenyl)-2-(4-sulfophenyl)-2H-tetrazolium, inner salt] (MTS). Cell viability was determined by measuring absorbance at 490 nm with a microplate reader (TECAN) and expressed as a percentage relative to control cells.

### Mitochondrial function assay

The Rod 123 dye distributes across the mitochondrial inner membrane in response to the negative membrane potential. When the function of mitochondrial is impaired, the membrane potential declines and mitochondria fails to retain Rod 123 [40]. MIN6 cells were seeded in 24-well plates and cultured to the confluence as detailed above. At the end of the 6-hour treatment period, cells were rinsed with PBS prior to incubation with Rod 123 (1 μg/ml) for 15 minutes at 37°C. Cells were dissociated and the fluorescence intensity was measured with a fluorescence microplate reader (FLUOstar OPTIMA, BMG LABTECH) at λex: 500 nm and λem: 530 nm. Results are expressed as relative fluorescence unit (RFU) normalized to protein content measured by BCA protein assay kits.

### Dihydroethidium (DHE) assay

DHE is a reduced fluorophore, which has been extensively used for mitochondrial superoxide detection [41]. MIN6 cells were seeded in 24-well plates and grown to 80~90% confluence. Cells were serum-starved for 6 hours prior to incubation with 20 μM DHE for 30 minutes at 37°C. Cells were then incubated with different treatments in the presence or absence of Hex (1 μM) and STZ (1 mM) for 6 hours. Cells were dissociated and the fluorescence intensity was measured with a fluorescence microplate reader (FLUOstar OPTIMA, BMG LABTECH) at λex: 470 nm and λem: 590 nm. Results are expressed as RFU normalized to protein content.

### Protein extraction and Western blot

Following the 6-hour incubation treatments, MIN6 cells were rinsed with ice-cold PBS and lysed in RIPA buffer (Cybrdi, Frederick, MD, USA) supplemented with a protease-inhibitor cocktail (Roche, Basel, Switzerland). Cell lysates were cleared by centrifugation at 12,000 rpm for 20 minutes at 4°C and total protein concentration was determined with the BCA protein assay kit (Pierce, Rockland, IL, USA). The protein (20 μg) of each sample was separated on 10% SDS-PAGE gel and electro-transferred to nitrocellulose membranes (Millipore Corporation, Bedford, MA, USA). Membranes were blocked for 1 hour with 5% non-fat milk in TBS-T buffer at room temperature prior to an overnight incubation with various primary antibodies at 4°C. The following primary antibodies were used (all at 1:1000 dilution): anti-Bax, anti-Bcl-2, anti-cleaved Caspase-3 (Cell Signalling Technology, Danvers, USA), anti-cleaved Caspase-9 and α-tubulin (Abcam, Cambridge, UK). Membranes were then washed and incubated with HRP-conjugated secondary antibody (GE Healthcare, Freiburg, Germany) (at 1:5000 dilution), followed by detection with enhanced chemiluminescence (Thermo scientific, Rockford, IL, USA). The membranes were then stripped and re-probed with α-tubulin to ensure equivalent loading and transfer of protein. Semi-quantitative densitometry analysis was performed on scanned films using ImageJ software.

### Animals

Experiments were performed in male Wistar rats (180–200 g), provided by the University of Queensland Biological Resources (UQBR). Rats were housed separately under standard housing conditions (lights on from 06:00 to 18:00, temperature 22 ± 2°C) and had access to food and water *ad libitum*. The experimental protocols were approved by the University of Queensland Animal Ethics Committee. A single intra peritoneal injection of STZ dissolved in a 0.1 mol/L citrate buffer (pH 4.5) was used at a dose of 65 mg/kg body weight. Age-matched control rats received an equal volume of vehicle (sodium citrate buffer). Four weeks after STZ or vehicle injection, rats were randomly assigned to 4 groups (n = 16/group) according to the treatment: rats with vehicle then saline for 2 weeks (control group); or 100 μg/kg Hex for 2 weeks (Hex treated group); STZ-treated rats then saline for 2 weeks (STZ treated group); or 100 μg/kg Hex for 2 weeks (STZ + Hex treated group). Saline and Hex were intra-peritoneally injected daily.

### Measurement of blood glucose and plasma insulin level in rat

At the end of STZ treatment, rats from each treatment group were randomly divided into 2 sub-groups; 8 of them were fasted overnight, and the other 8 rats were fed as normal. Blood glucose level was measured with terminal blood samples by Accu-Chek glucometer (Roche, Indianapolis, IN). The samples were collected using a heparinised syringe (100 IU/ml) by cardiac punch from anesthetized rats. Plasma samples were immediately collected *via* centrifugation (6000 rpm for 3 min). Fasting and fed circulating insulin levels were determined by commercial insulin ELISA kits (EZRMI-13K rat/mouse insulin, Millipore, Billerica, MA).

### Immunofluorescence staining of rat pancreatic islets

Rat pancreas were fixed in 4% paraformaldehyde at 4°C overnight, followed by immersion in 30% sucrose for 3 days at 4°C, frozen, and cut on a cryostat. To measure surviving cell types and apoptosis, pancreatic sections (30 μm) were incubated overnight with the following primary antibodies: mouse anti-insulin (1:400) (Millipore Corporation, Bedford, USA), rabbit anti-glucagon (1:400) (Thermo Scientific, Rockford, USA), rabbit anti-Bax (1:400) (Cell Signalling Technology, Danvers, USA) and rabbit anti-Caspase-9 (1:100) (Abcam, Cambridge, UK). Secondary antibodies conjugated with Alexa 546 or Alexa 488 (1:2000; Invitrogen, Melbourne, Australia) were used as appropriate. ProLong Golden anti-fade mounting reagent with DAPI (Invitrogen, Melbourne, Australia) was added onto the slides to counterstain the nucleus and preserve the fluorescent signals. Images were acquired and compiled using confocal microscopy (LSM 510 META, Zeiss) for quantitative analysis.

Imaging quantification was performed by Imaris (Bitplane, Zurich, Switzerland). The number of insulin- and glucagon-positive cells and the diameter of isolated islets were measured. Quantitative data were collected from eight randomly selected sections in 8 to 12 islets of each rat [42]. Cells positively stained with Bax and Caspase-9 were counted and quantified relative to total cell numbers (DAPI-stained). Results are expressed as percentage relative to the control group.

### Statistical analysis

Data are expressed as mean ± standard error of mean (SEM). Statistical significance was determined using one- or two-way analysis of variance (ANOVA) followed by Dunnet post hoc tests. The F and *P* values for each main factor were reported. A *P* value <0.05 was considered as statistically significant. Analyses were conducted by GraphPad software Prism 5.0 (GraphPad Software, San Diego, CA, USA).

## Results

### Effect of STZ and Hex on the viability of MIN6 cells

**Fig 1:**
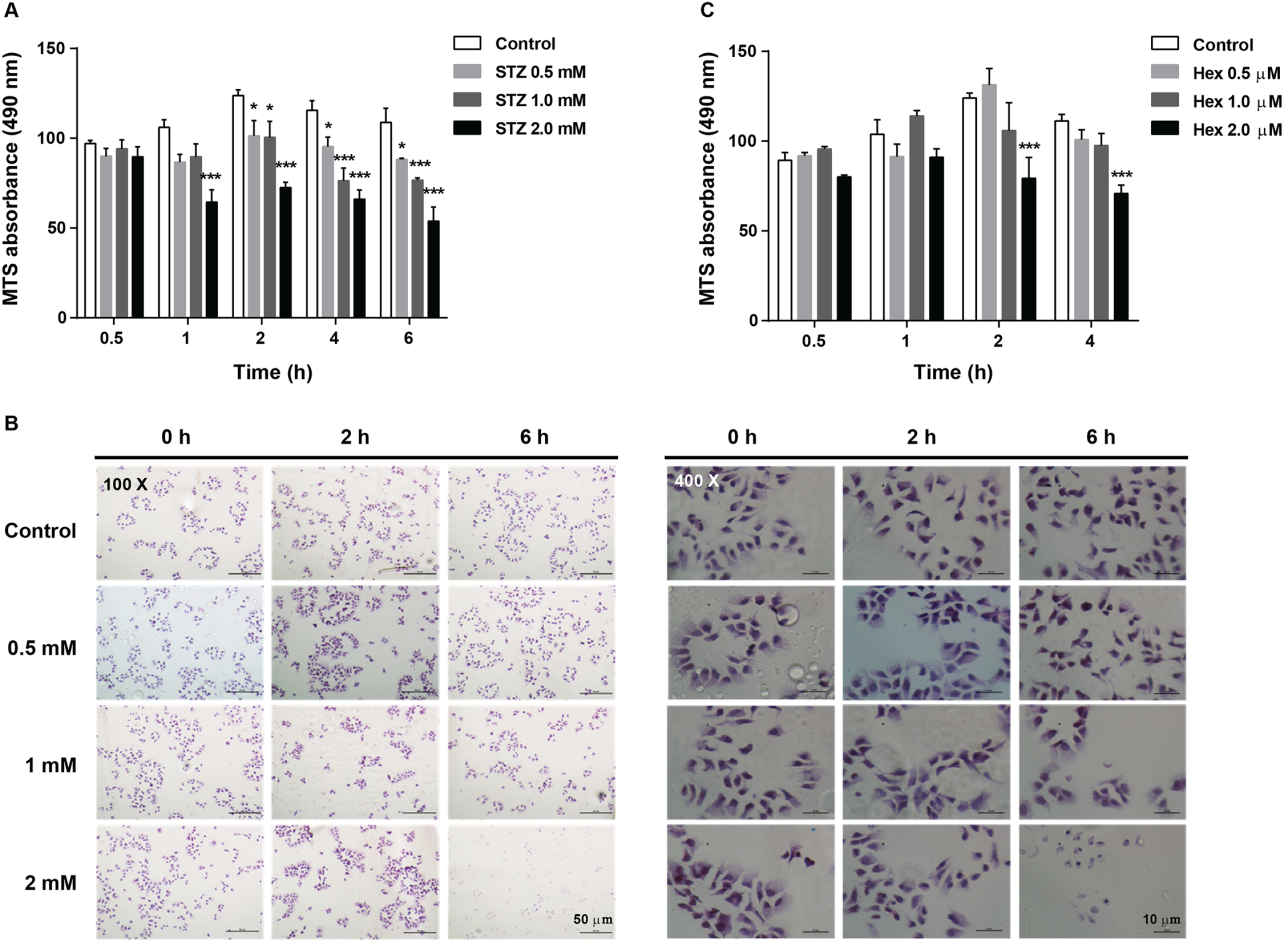
STZ induced time- and dose-dependent cytoxic effect in MIN6 cells. (A) The viability of MIN6 cells treated with STZ (0.5, 1.0 and 2.0 mM) for various time periods (n = 6). (B) The representative images of STZ-treated MIN6 cells at a magnification of 100× and 400×, respectively. MIN6 cells were stained with Hematoxylin-Erosin (HE). (C) The viability of MIN6 cells treated with Hex (0.5, 1.0 and 2.0 μM) for various time periods (n = 6). The data is presented as the mean ± SEM. *p < 0.05, **p < 0.01, ***p < 0.001, compared with the corresponding control group at each time point.

The viability of MIN6 cells was significantly decreased following STZ treatment in a dose- and time-dependent manner (F_dose (3, 8)_ = 29.34, *P* < 0.001; F_time (4, 32)_ = 6.313, *P* < 0.01; F_interaction (12, 32)_ = 2.960, *P* < 0.01) (Fig 1A). Treatment the cells with 2 mM STZ for 1 hour led to a significant reduction in the cell viability (*P* < 0.001). Further increase in the incubation time resulted in a more pronounced reduction, which reached the lowest value after a 6 h incubation with 2 mM STZ (*P* < 0.001). To further investigate the observed deleterious effect of STZ on MIN6, morphological changes in MIN6 cells were demonstrated by H&E staining after 0, 2 and 6 h of STZ treatment. As showed in Fig 1B, increased STZ dose and incubation time resulted in severer reduction of MIN6 population. Most strikingly, there was hardly any viable MIN6 cells after 6 h incubation of 2.0 mM STZ (40×). The cytotoxic effect of Hex was also observed in the cells with a high concentration (2 μM) for incubation time 2 and 6 hours (F_dose (3, 8)_ = 12.49, *P* < 0.01; F_time (3, 24)_ = 6.815, *P* < 0.01; F_interaction (9, 24)_ = 2.827, *P* < 0.05) (Fig 1C), whereas lower concentrations of Hex had no obvious effect. Based on these data, an optimal treatment strategy of 1.0 mM STZ for 4 hours followed by 1 μM Hex for 2 hours was selected for MIN6 cells in the subsequent experiments.

### Protective effect of Hex on STZ-treated MIN6 cells

**Fig 2:**
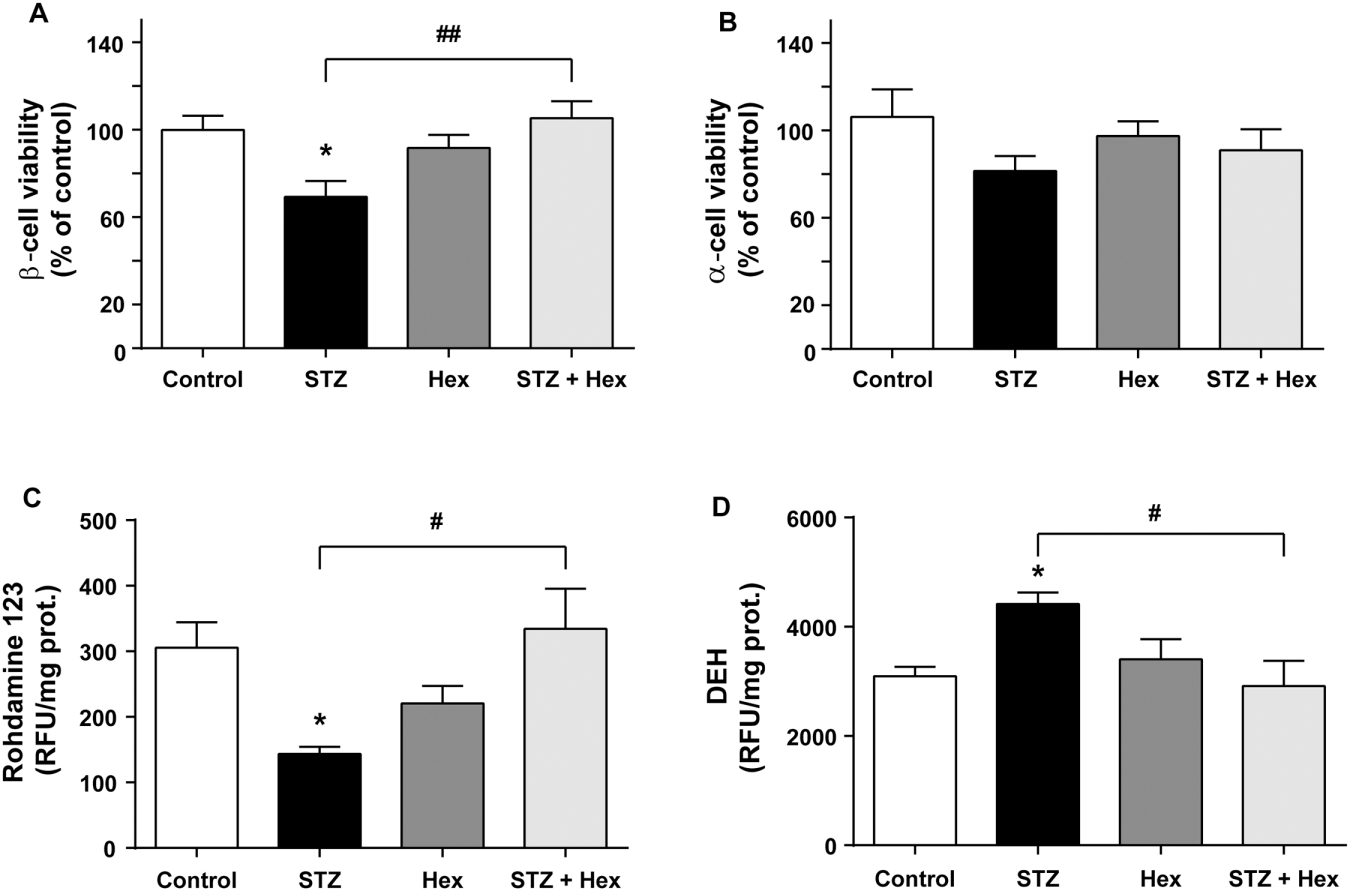
Hex reduced STZ-induced mitochondrial damage and in pancreatic cells. The MIN6 and α-TC6 cells were treated with 1.0 mM STZ and 1.0 μM Hex alone or in combination. Cell viability of (A) the MIN6 and (B) α-TC6 cells was measured by MTS assay (n = 6). The MIN6 cells were then treated with 1.0 mM STZ and 1.0 μM Hex alone or in combination. (C) The changes in the mitochondrial potential of the MIN6 were determined by Rhodamine 123 assay. (D) Cell superoxide activity of the MIN6 was detected by superoxide DHE production. The data are presented as the mean ± SEM (n = 8). *p < 0.05, compared with the control group; #p < 0.05, ##p < 0.01, compared with the STZ group.

Cultured MIN6 cells were incubated with SFM (control), 1 μM Hex, 1 mM STZ alone or STZ followed by Hex for 6 hours. The viability of MIN6 was significantly affected by different treatments of the cells (F_(3, 36)_ = 5.244, *P* < 0.01) (Fig 2A). As expected, STZ alone induced a significant growth reduction as compared with the control cells (69.3% of control, *P* < 0.001), whereas Hex alone did not show any notable effect. MIN6 cells treated with STZ followed by Hex received a great increase in the cell viability (105.3% of control, *P* < 0.01) as compared with those treated with STZ alone. Noteworthy, we also examined the effects of STZ and Hex on α-TC6 cells, a mouse pancreatic α-cell line (Fig 2B). STZ and/or Hex treatment did not significantly affect the viability of α-TC6 cells (F_treatment (3, 30)_ = 1.313, *P* = 0.2936).

### Protective effect of Hex on maintaining mitochondrial function and against oxidative stress in STZ-treated MIN6 cells

It is known that STZ can damage β-cells *via* its harmful effect on mitochondrial function [4, 34]. In order to examine the protective effect of Hex on STZ-induced mitochondrial damage, the mitochondrial function was determined by Rod 123 assay (F_(3, 27)_ = 3.619, *P* < 0.05) (Fig 2C). The ratio of RFU against protein content (mg) for STZ-treated cells was significantly reduced in comparison to the control cells (*P* < 0.05), indicating impairment in mitochondrial function. Furthermore, the RFU/mg ratio in the STZ (1 mM) treated MIN6 cells was significantly lower than those treated with STZ+Hex (*P* < 0.05), suggesting that Hex protects the cells from STZ-induced mitochondrial damage. The protective effect of Hex was further verified by superoxide production DHE assay (F_(3, 47)_ = 4.246, *P* < 0.05) (Fig 2D). The DHE levels in STZ-treated cells were significantly elevated (*P* < 0.05), but were markedly reduced by further treated with Hex as indicated by the ratio of RFU to protein content (*P* < 0.05). Taken together, these results demonstrate that Hex protects β-cells from STZ-induced oxidative stress, which leads to mitochondrial dysfunction.

### Influence of Hex on protein levels of Bax/Bcl-2 and cleaved Caspase-3 and -9

**Fig 3:**
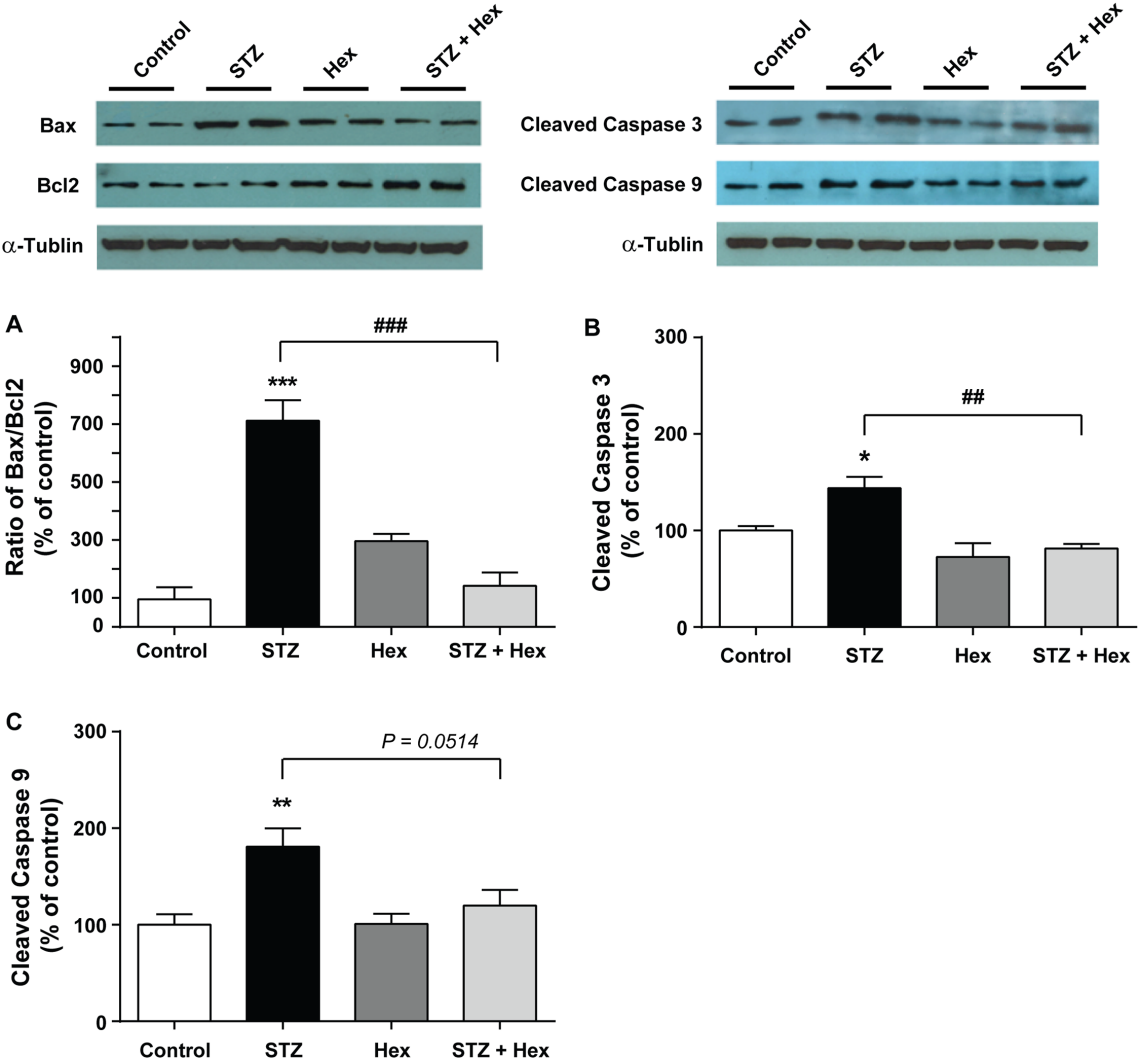
Expression of Bax/Bcl-2 and activated Caspase 3 and 9 caused by STZ and Hex. The MIN6 cells were treated with 1.0 mM STZ and/or 1.0 μM Hex, the alterations of Bax/Bcl-2, cleaved Caspase-3 and cleaved Caspase-9 were detected by Western blot. The upper panels show the representative immunoblots for each protein. (A) Hex significantly decreased the ratio of Bax to Bcl-2 increased by STZ treatment. (B and C) Hex significantly decreased the STZ-induced expression of cleaved Caspase-3 and cleaved Caspase-9. The data are presented as the mean ± SEM (n = 4). *p < 0.05, **p < 0.01, ***p < 0.001, compared with the control group; ##p < 0.01, ###p < 0.001, compared with the STZ group.

In addition to oxidation and ATP production, mitochondria also play a crucial role in the process of cell apoptosis. STZ-treated MIN6 cells showed a significant increase in the ratio of Bax/Bcl-2 (F_(3, 15)_ = 12.14, *P*<0.001) (Fig 3A), and also in the protein expression of cleaved Caspase-3 (F_(3, 14)_ = 33.49, *P*<0.001) (Fig 3B) and cleaved Caspase-9 (F_(3, 18)_ = 12.14, *P*<0.01) (Fig 3C), as compared to expression of these proteins in the control group. The ratio of Bax/Bcl-2 (*P*<0.001), and cleaved Caspase-3 (*P*<0.01) and cleaved Caspase-9 (*P* = 0.05) protein levels were significantly declined in cells treated with STZ followed by Hex, as compared to STZ alone. These results indicate that Hex may counteract the apoptotic effect induced by STZ on MIN6 cells.

### Blood glucose level and insulin secretion in STZ- and Hex-treated rats

**Fig 4:**
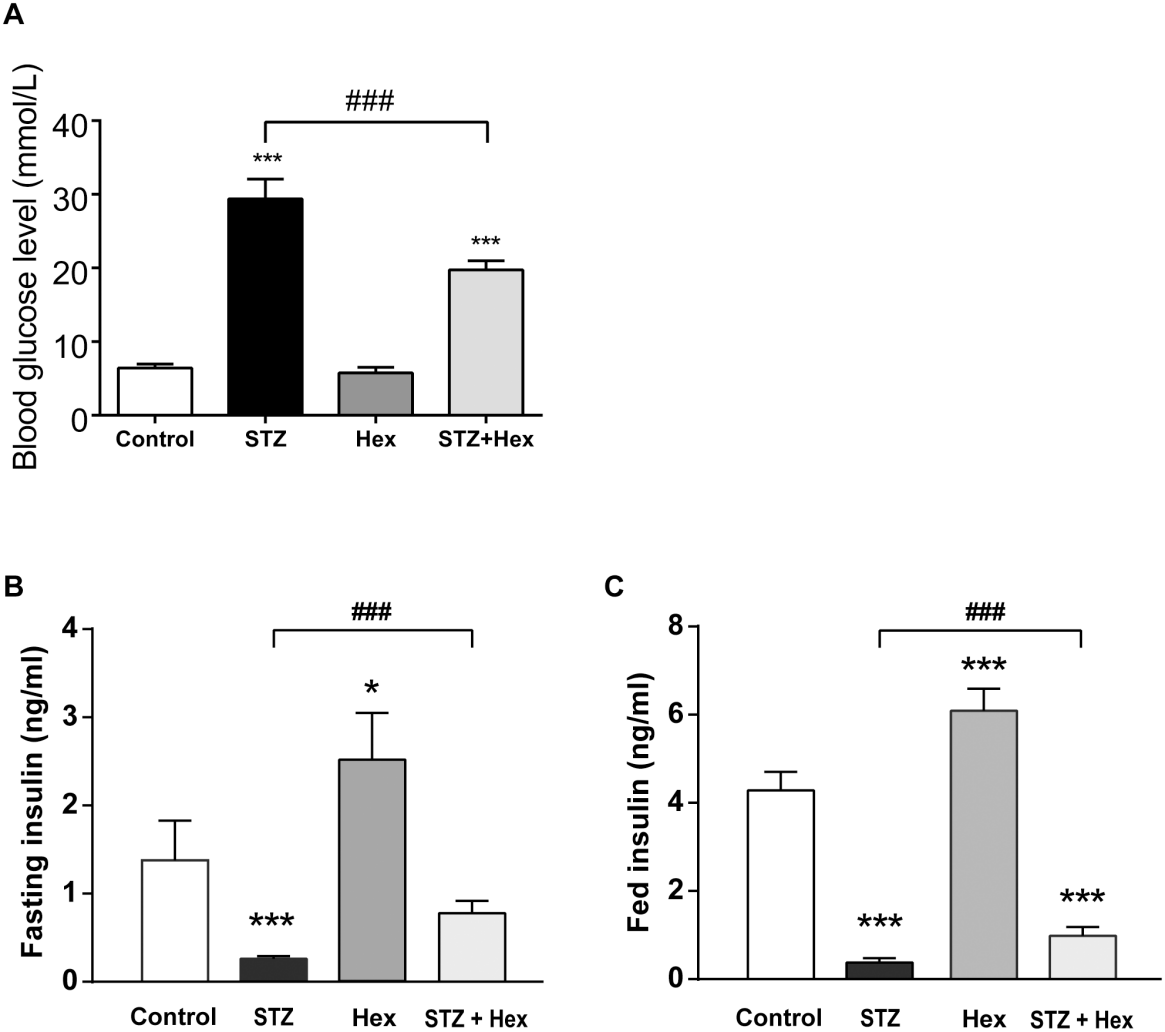
Blood glucose level and fasting or fed plasma insulin levels from STZ- and/or Hex-treated rats. (A) After the STZ or Hex treatment, blood glucose level was measured with Accu-Chek glucometer after overnight fasting (n = 16). Three days after measurement of blood glucose level, half of rats (n = 8) were fasted overnight, and the other half were fed as normal. Terminal blood samples were collected. Plasma insulin levels from (B) fasting or (C) fed rats were determined by insulin ELISA kits. The data were presented as the mean ± SEM. *p < 0.05, **p < 0.01, ***p < 0.001, compared with the citrate + saline group; ### p < 0.001 between the two indicated groups.

Since the *in vitro* experiments showed that Hex is likely to exert a protective effect against STZ-induced cytotoxic effects on β-cells, we further examined the *in vivo* effect of Hex (100 μg/kg) in STZ-induced diabetic rats. STZ-injection dramatically increased blood glucose levels in rats whereas Hex treatment diminished the effect of STZ (Fig 4A). Plasma insulin levels of rats were measured under both fasting and fed conditions respectively. Different treatment exerted significant effects on fasting insulin level (F_(3, 28)_ = 7.472, *P*<0.01) (Fig 4B). The Hex alone increased the plasma insulin (*P*<0.05) whereas the STZ dramatically decreased it (*P*<0.001). STZ + Hex-treated rats showed an increased insulin level (*P*<0.001). Similar to fasting insulin level, fed insulin level (F_treatment (3, 28)_ = 61.89, *P*<0.001) were significantly increased in Hex-treated rats (*P* < 0.001) and reduced in STZ-treated rats (Fig 4C). Furthermore, the Hex also ameliorated STZ-induced decrease in fed plasma insulin level (STZ *vs*. Hex + STZ, *P*<0.001).

### Islet cell quantification in STZ- and Hex-treated rats

Immunohistochemistry was performed to identify subpopulations of α- and β-cells in islets from rats with various treatments as above. In general, the islets were round, with a clear boundary, normal structure of central β-cells surrounded by α-cells in control rats (data not shown). In the STZ-treated rat, destructive islets were observed with dramatically declined number of β-cells, while the proportion of α-cells seemed increased as some were located within the central portion of islets. In the group treated with Hex after STZ injection, the loss of β-cells was significantly less than observed for STZ injection alone and the structure of islets was closer to normal.

**Fig 5:**
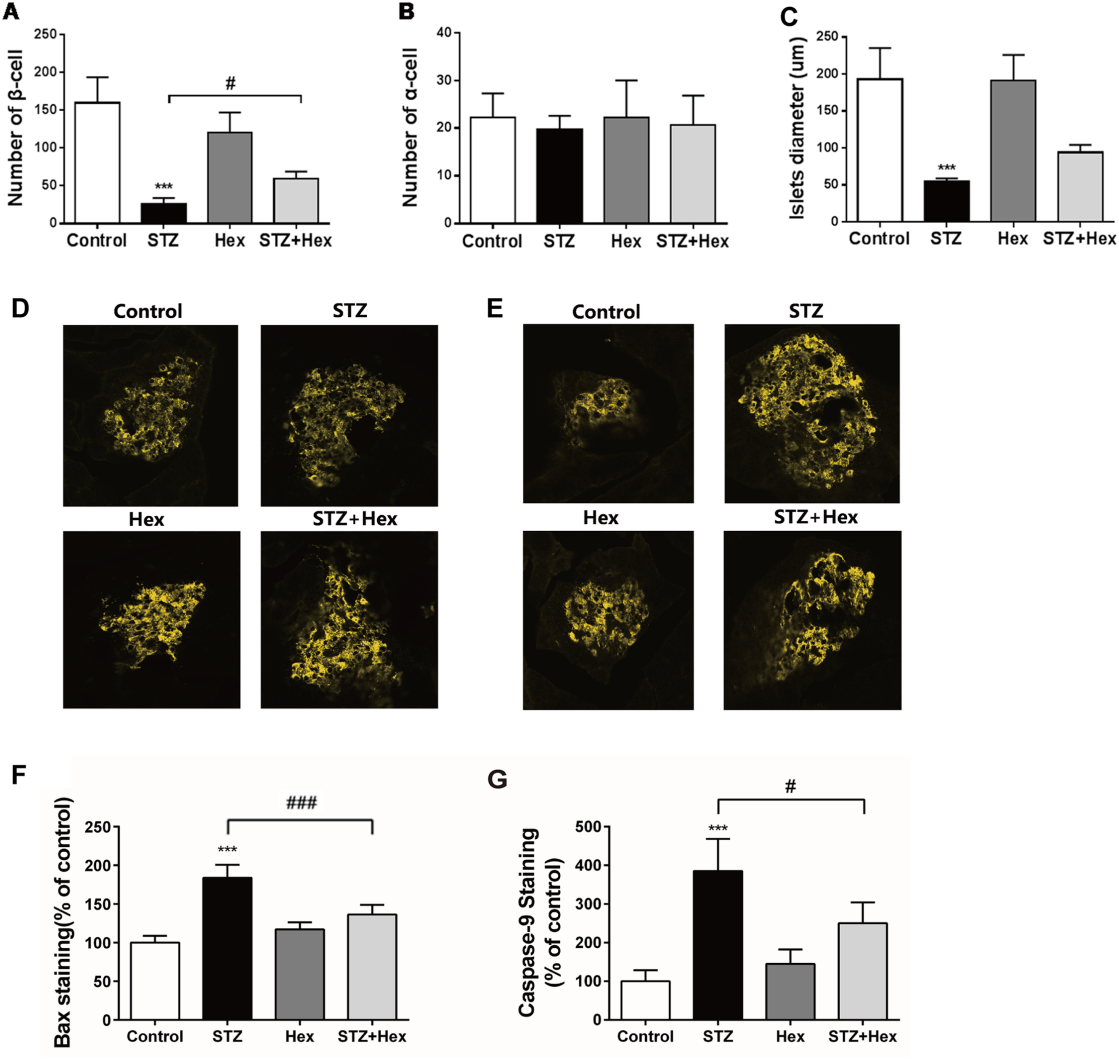
Quantification of glucagon and insulin immunostaining of islet and quantification of Bax and Caspase-9 immunostaining in STZ- and/or Hex-treated rats. After image quantification, (A) number of β-cell and (B) α-cell, (C) diameter of pancreatic islets were calculated from rats after STZ and/or Hex treatment. Representative histological image of pancreas sections from rats after STZ and/or Hex treatment were also showed. The sections were immunofluorescent stained with (D) Bax and (E) Caspase-9 antibody and observed under confocal microscopy. After image quantification, the percentage of (F) Bax and (G) Caspase-9 staining of islets were calculated from rats after STZ and/or Hex treatment. The data were presented as the mean ± SEM (n = 4). ***p < 0.001, compared with the citrate + saline group; #p < 0.05 between the two indicated groups.

The population of β-cell per islet (average of 10 islets randomly selected from slices per rat) in STZ-treated rats was significantly reduced in comparison to the control group (*P* < 0.001) (Fig 5A). Moreover, the number of β-cells in the animals treated with STZ was significantly lower than the STZ+Hex group (*P* < 0.05). There was no significant difference on the number of α-cells among groups (Fig 5B). Due to a loss of β-cell numbers in the islets of STZ-treated rats, the size of islet in these animals was significantly reduced in diameter in comparison to the control group (*P* < 0.001) (Fig 5C). Therefore, it is suggested that Hex may protects the size and structure of islet from STZ injection *in vivo*.

### Signalling molecule changes in islets from STZ- and Hex-treated rats

As indicated in Fig 5D and 5E, pancreatic islets from treated rats were detected for Bax and Caspase-9 protein expression. The number of Bax-positive cells in the STZ-treated animals was 70% higher when compared to the control animals, whereas further treatment with Hex (STZ+Hex) showed significant decrease in Bax compared with STZ-treated rats. There was a significant increase in Caspase-9 positive cells in the islets isolated from STZ-treated rats in comparison to the control group (Fig 5E). Furthermore, the number of Caspase-9 positive cells was also significantly decreased in “STZ+Hex” co-treated rats. These data suggest that Hex administrated at an appropriate dose may protect β-cells *in vivo* from STZ-induced damage.

## Discussion

We have provided *in vitro* and *in vivo* evidence that Hex protects β-cell mitochondrial functions from STZ-induced damage. Hex is a synthetic peptide that shows similar biological properties to ghrelin. Our previous studies showed that Hex protected cardiomyocytes from ischemic damage [36, 37]. A high dose (2 mM) of STZ induced apoptosis in MIN6 β-cells *in vitro*, which was further verified by the *in vivo* results of STZ-induced diabetic rats, where Hex showed beneficial effects on pancreatic islets. *In vitro*, STZ decreased β-cell viability and Hex offset this effect.

The STZ model is often used to selectively damage pancreatic β-cells *in vivo* and *in vitro*. Many agents have been tested in this model such as phytochemicals and cytokines [42, 43]. The STZ model is also used to demonstrate protective function of intracellular proteins by over-expression them in β-cells, such as SDF-1/CXCL12/Akt system [44] and Reg-2 [45]. We used this system to test the effect of hexarelin in this experiment.

Previous studies have demonstrated that insulin secretion may be suppressed by ghrelin, and pharmacological blockade of ghrelin maybe used to treat type 2 diabetes [46]. However, there is also evidence showing that overexpression of ghrelin promotes proliferation and inhibits apoptosis of β-cells after STZ-induced injury [47, 48]. In this experiment, appropriate dose (1 μM) of Hex, a synthetic analogue of ghrelin, protected MIN6 cells from cytotoxic effect of STZ.

Apoptosis is a process of programmed cell death, which is closely associated with β-cell mass in diabetes. Mitochondrial signalling systems direct cells to apoptosis *via* Bcl-2 family [49], through the increases in cleaved Caspase-3 and -9. Jian *et al*. showed that overexpression of adiponectin in mice reduced STZ-induced apoptosis through Caspases-3, -8 and -9 [50]. We have shown here that STZ increases the ratio of Bax to Bcl-2 while Hex after STZ restores this ratio. It is suggested that Hex protects β-cells from STZ-induced apoptosis through the protection of mitochondrial functions. This was further demonstrated by the findings that Hex suppresses STZ-induced activation of Caspase-3 and -9, loss of mitochondrial membrane potential and excessive superoxide production.

The *in vivo* protective effect of Hex against cytotoxic effect of STZ on β-cells was further supported in rat diabetic model by STZ injection. Hex (100 μg/kg) ameliorates STZ-decreased plasma insulin in rats, which is most likely to result from protection of β-cells by Hex. Immunohistological examination of pancreatic islets in this experiment has reinforced the notion that Hex protects β-cells *in vivo* from STZ-induced β-cell death.

## Conclusion

This study demonstrates that Hex is able to protect β-cell mass from STZ-caused cytotoxic effects involving mitochondrial pathways *in vitro* and *in vivo*. Minimal side effects of Hex have been observed in clinic trials, and the fabrication of this small peptide of 6-amino acids is economic. Hex represents a promising anti-diabetic drug to protect pancreatic islets.
